# The effect of social media environmental information exposure on the intention to participate in pro-environmental behavior

**DOI:** 10.1371/journal.pone.0294577

**Published:** 2023-11-16

**Authors:** Yanfang Meng, Donghwa Chung, Anxun Zhang

**Affiliations:** 1 School of Journalism and Communication, Beijing Institute of Graphic Communication, Beijing, People’s Republic of China; 2 School of Journalism and Communication, Central China Normal University, Wuhan, People’s Republic of China; Universiti Kebangsaan Malaysia, MALAYSIA

## Abstract

With the threat of global warming, countries worldwide have enhanced their environmental campaigns on social media to increase users’ willingness to take pro-environmental actions. In this study, we examined the direct and indirect effects of exposure to environmental information on Chinese young adults’ (18–25 years old) intention to participate in environmental protection actions (e.g., recycling, using public transportation, involvement in an environmental group, and participation in eco-friendly events). Data were collected from a sample of 291 Chinese young adults using a web-based survey and a thoroughly designed questionnaire. The accumulated data were analyzed using SPSS version 20. Hierarchical regression and mediation analysis were performed for testing hypotheses. The results indicated that exposure to environmental information on Chinese social media platforms (WeChat and Xiaohongshu) positively affected individuals’ intention to participate in pro-environmental behavior, perceived pro-environmental behavior control, pro-environmental attitude, and fear of victimization. The indirect effect demonstrated that pro-environmental behavior control and attitude mediated the relationship between exposure to environmental information on both WeChat and Xiaohongshu and the intention to participate in pro-environmental behavior. Extending the existing literature, this study provides empirical evidence on the influence of environmental information exposure on the intention to participate in environmental protection among Chinese adults. In addition, it provides valuable insights into the mediating mechanisms involving cognitive, psychological, and emotional factors in this relationship. Policy makers should implement effective pro-environmental promotions on social media to inspire individuals to engage in environmentally friendly actions. In addition, social media managers should strictly authenticate and remove misleading environmental content.

## Introduction

In recent years, the negative consequences of global warming have raised concerns. In China, smog has plagued many cities year-round, with frequent rainstorms and floods [1]. A range of impacts caused by climate change, such as the El Niño and La Niña phenomena, have disrupted individuals’ daily lives and strengthened public attention to environmental threats [2]. However, in most cases, such environmental issues are rooted in human behavior [3]. The production and consumption of methane (CH4) and carbon dioxide (CO2) are the main factors leading to the uneven distribution of greenhouse gas (GHG) fluxes, often referred to as "hot spots" or "hot moments" [4]. Current evidence suggests that China has become one of the world’s largest emitters of carbon dioxide, accounting for about 30% of global carbon emissions [5]. Evidence firmly claimed that China as the world’s leading energy producer, with coal and hydropower making significant contributions to its electricity generation of 7,503 TWh in 2019. Since 2009, China has consistently accounted for about 28% of global CO2 emissions [6]. Among Chinese provinces, Shanxi’s CH4 emissions significantly exceed those of other provinces, accounting for 35.5% of the country’s total emissions. Chongqing also stands out as having the highest emissions from coal mining in China, at approximately 60.9 m3/t [7]. Despite China’s historically high production and consumption of CH4 and CO2, a latest study reveals a positive shift in their approach to environmental responsibility. For instance, According to Wang et al. [8], Chinese efforts to control coal mine methane have effectively ensured safe coal mine production since 2005. This has resulted in the substitution of 53.21 million tons of standard coal consumption and a reduction in greenhouse gas emissions of 958.55 TgCO2e. In response to these problems, scientists and policymakers suggest that these problems can be mitigated by increasing individuals’ awareness of climate change and their willingness to participate in pro-environmental behaviors [9–11].

Pro-environmental behavior is defined as the effort to minimize the negative effects of actions on the natural and built worlds [12]. In this study, intention to participate in pro-environmental behavior (IPPEB) refers to individuals’ willingness to participate in all possible actions aimed at safeguarding the environment, either by participating in private (e.g., recycling, using public transportation, and eco-friendly purchasing) or public spheres (e.g., involvement in an environmental group or participation in eco-friendly events) [13, 14]. Previous studies have found that social media platforms provide great opportunity to engage in environmental education [15]. Specifically, this has increased individuals’ awareness of the current environmental status and how it has an impact on society [16]. This has further instigated social media users’ IPPEB [17]. Despite the fact that earlier studies have concentrated on the relationship between exposure to climate-change-related information on social media (e.g., Facebook and Twitter) and the degree of individuals’ IPPEB [18–20], the great majority of studies have investigated the effect of media exposure on environmental-related behaviors in western countries and southeast Asian countries [18, 21]. However, whether exposure to such information on Chinese social media significantly affects social media users’ IPPEB is currently unknown.

In recent years, China’s rapid economic growth and increased material production have had an impact on natural resource consumption and environmental pollution [22]. Such an impact has given rise to one of the nation’s most urgent and severe challenges. In response to this phenomenon, the Chinese government has been focusing on effective ways to reduce ecological degradation [23]. For instance, in 2023, the Chinese government implemented an innovative strategy which is known as the "Four Water and Four Determination" initiative. Such a strategic approach is focused on effectively addressing water-related challenges in the country. Its primary objectives are to strictly prevent sewage discharge into rivers or seas, promote individuals’ responsibility of water consumption, and reduce household overall water waste. This initiative particularly stressed that farmers should adopt water-saving practices. By doing so, the government aims to ensure sustainable agricultural water usage while also keeping a strict check on the total volume of water utilized in the agricultural sector. The initiative has gained significant attention from industries and institutes with its strong emphasis on water conservation and addressing environmental concerns. On 27 March 2023, the China Annual Conference on Environmental Communication reported that launching an effective promotion campaign on social media should be considered as one strategy for environmental protection. Particularly, they suggested that increasing the dissemination of protective and environmental-degradation-related content, such as news, scientists’ presentations, documentaries, and educational materials on social media platforms (e.g., WeChat and Weibo), will enhance individuals’ environmental awareness [24].

Previous studies have investigated some key factors that positively affect individuals’ pro-environmental behaviors. For instance, the length of time spent on the Internet [25], satisfaction with government environmental protection [26], and education level [27] have been shown to increase IPPEB. However, previous studies have argued that the process of being exposed to environmental information on social media platforms to experiencing IPPEB is complex, as behaviors are not only the result of a single social factor or demographic variable, but are also affected by a combination of various cognitive, psychological, and emotional factors [12, 28, 29]. To the best of our knowledge, previous studies have failed to investigate issues of how effectively pro-environmental communication works on some Chinese mainstream social media platforms, how exposure to environmental information on social media platforms may promote individuals’ IPPEB, and what the key mediating mechanisms are in such a relationship. Investigating such a phenomenon provides critical contributions to our understanding of the effects of Chinese social media platforms on individuals’ IPPEB.

Focusing on the Chinese context, this study is guided by the theory of planned behavior (TPB), which aims to investigate the process of exposure to environmental information through various Chinese social media platforms on the IPPEB among young adult users (18–25 years old). Therefore, the effects of cognitive, psychological, and emotional factors are considered, such as perceived behavioral control, attitude, and fear of victimization (FV).

## Theoretical background and literature review TPB

The TPB was expanded by Ajzen based on the theory of reasoned action (TRA). This theory found that “people do not always act voluntarily but out of some kind of control” and defined this phenomenon as perceived behavioral control [30]. The TPB explains how human attitudes, subjective norms, and perceived behavioral control change human intentions, and thus change final behavior [30]. To better understand the mechanisms of conscious decision-making, numerous scholars have applied the TPB in many studies across disciplines, such as environmental issues [31] and health behavior in epidemic circumstances [32]. However, the TPB has been criticized for failing to explain certain behavioral changes owing to emotional, environmental, and cultural factors [33]. Additionally, some studies have found that the original components of the TPB (such as subjective norms) have weaker predictive power for behaviors [34]. In response to such criticisms, previous studies have extended the TPB model to improve its explanatory capacity [35]. By including additional explanatory variables in the original TPB model, it became widely accepted in a number of studies. For instance, Ajzen indicates that the TPB, in principle, is open to the inclusion of additional explanatory predictors, as long as they provide facts that are significant and contribute to the theory’s development [30]. Moreover, extended TPB models have shown superior predictive abilities compared to the original model. Specifically, this has increased the level of explanatory power of individuals’ intentions to engage in certain types of behavior (e.g., adopting electric vehicles, consuming genetically modified foods, and the intention of travelers to visit countries) [35–37].

In this study, to better understand the relationship between environmental information exposure on social media platforms and Chinese young adults’ IPPEB, subjective norms are omitted from the original model. Instead, FV is considered an extended component that has been added to the extended TPB model, as many studies have stressed that provoked fear induces IPPEB [38, 39]. Therefore, this may be a key determinant of IPPEB in Chinese young adults. The theoretical framework of the current study is expected to provide a better understanding of such a phenomenon and further explore the effects of each component on Chinese young adults’ IPPEB.

### Effects of information exposure on cognitive, psychological, and emotional factors

Among instant messaging applications, Tencent’s WeChat is a powerful social application. Its users span 31 regions and 194 countries, with a total user base of more than 1.2 billion [40]. WeChat’s main functions include sending text and voice messages, sharing personal updates and locations, mobile payments, and reading articles published by WeChat’s official accounts, which can be applied to most scenarios in daily life [41]. While in the field of e-commerce, with the integration of social media and the e-commerce industry, Xiaohongshu (also known as Little Red Book) has become an emerging popular software among Chinese young adult groups. According to an analysis of e-commerce industry data, in November 2020, Xiaohongshu had 12,321,900 active monthly users [42]. As of November 2021, Xiaohongshu had 300 million users. On the Xiaohongshu platform, users build social relationships by sharing posts, browsing the content posted by others, and engaging in cross-border consumption [43]. In China, WeChat stands out because it reinforces individual-centered social relationships, whereas Xiaohongshu provides higher-quality information and reflects the outward expansion of social relationships. As a mainstream communication application in China built upon solid social relations, WeChat’s user satisfaction is crucial for measuring users’ willingness to continue using it [44].

A previous study conducted a survey on WeChat users’ information exposure. The results indicated that these users were generally satisfied with the timeliness and clarity of their information. However, they were dissatisfied with its non-authenticity and diversity [45]. In addition, WeChat can provide the latest information that helps users keep up with current social events [46]. However, false information and excessive notifications affect information quality. Compared with WeChat, the information-sharing application, Xiaohongshu, emphasizes the exchange and acquisition of information and weakens personal social relationships. These include social media, sharing platforms, and software services [47]. Although Xiaohongshu encourages users to share information and improve their engagement through various marketing activities, user surveys have shown that users have a negative attitude toward its notification mechanism and content censorship [48]. As previously discussed, WeChat and Xiaohongshu have advantages and disadvantages, leading to different intentions after users access the information on the two platforms. Therefore, based on the current situation of mainstream social media in China, it is critical to compare exposure to environmental information on WeChat (EEIW) and exposure to environmental information on Xiaohongshu (EEIX). Moreover, this study aimed to explore how users’ IPPEB changed after they were exposed to information on the two platforms.

Perceived behavioral control is recognized as a strong cognitive factor in individuals’ expectancy that the performance of the behavior is within his/her control [49]. This refers to individuals’ perception of the difficulty of a specific behavior [50]. In this study, perceived pro-environmental behavior control (PPEBC) is defined as individuals’ perceptions of the difficulty of adapting to pro-environmental behaviors. Attitude is a psychological factor that affects individuals’ positive or negative evaluations of engaging in a specific behavior [30]. Prior studies indicated that the more positive the attitude toward a behavior, the stronger the intention to engage in environment-protecting behaviors (such as lake protection) [51]. In this study, pro-environmental attitude (PEA) refers to an individual’s concern for the natural environment and reflects common attitudes and opinions toward the ecological environment [52]. FV refers to a negative emotional response when individuals experience or perceive a threat [17]. Such emotional factors may stimulate an individual’s idea or willingness to engage in eco-friendly behaviors [17, 53]. In this study, FV was defined as fear caused by individuals’ awareness of the negative effects on the environment after being exposed to information about global warming.

Previous studies have demonstrated that information exposure on social media can encourage individuals to adopt eco-friendly behaviors [54, 55]. A study on health information suggests that frequent social media exposure enhances college students’ perceived behavioral control for receiving or completing HPV (human papillomavirus) vaccination [49]. Additionally, a previous study indicated that exposure to pro-environmental messages improved individuals’ attitudes [56]. In addition to investigating the relationship between information exposure and cognitive and psychological factors, a previous study explored how media affected individuals’ emotional changes in the environmental context. A previous study found that individuals exposed to higher levels of environmental threat information positively affected fear of climate change (FV) [57]. Thus, the following hypotheses are proposed:

H1: EEIW has a significant positive effect on (a) IPPEB, (b) PPEBC, (c) PEA, and (d) FV.
H2: EEIX has a significant positive effect on (a) IPPEB, (b) PPEBC, (c) PEA, and (d) FV.

### PPEBC’s role as a mediator

Studies have confirmed that social media use has a direct effect on an individual’s perceived behavioral control [58]. Perceived behavioral control is positively associated with individuals’ intention to adopt recommended health behaviors [59]. Similarly, the mediating role of perceived behavioral control factors has been verified in several studies. For example, an empirical environmental study found that people’s exposure to environmental media content enhances their perceived behavioral control, which in turn induces their intention to engage in pro-environmental behaviors [60]. In addition, a previous study found that exposure to information on social media enhances college students’ perceived HPV behavioral control, which increases their intention to receive the HPV vaccine [49]. It is logical that individuals who are exposed to environmental information on social media and perceive fewer obstacles to adopting environmental protection behaviors will enhance their pro-environmental conviction and be more likely to engage in pro-environmental behaviors. Thus, the following hypotheses are proposed:

H3a: PPEBC mediates the relationship between EEIW and IPPEB.
H3b: PPEBC mediates the relationship between EEIX and IPPEB.

### PEA’s role as a mediator

A previous study demonstrated that higher exposure to environmental information had a positive effect on concern for the natural environment and showed common attitudes toward environmental protection [56]. Ultimately, this has increased the use of public transit, cycling, and walking (in the private sphere) [61]. In addition, when individuals are exposed to issues related to climate change, it induces their PEA [62]. Moreover, another study suggests that PEA positively affects individuals’ daily task-related pro-environmental behaviors [63]. Similarly, exposure to environmental protection-related information, such as avoiding excessive energy consumption, improves individuals’ attitudes toward climate issues [64]. Improved attitudes further encourage individuals’ engagement in pro-environmental purchase behavior (the private sphere) [65]. Thus, the following hypotheses are proposed:

H4a: PEA mediates the relationship between EEIW and IPPEB.
H4b: PEA mediates the relationship between EEIX and IPPEB.

### FV’ role as a mediator

**Fig 1:**
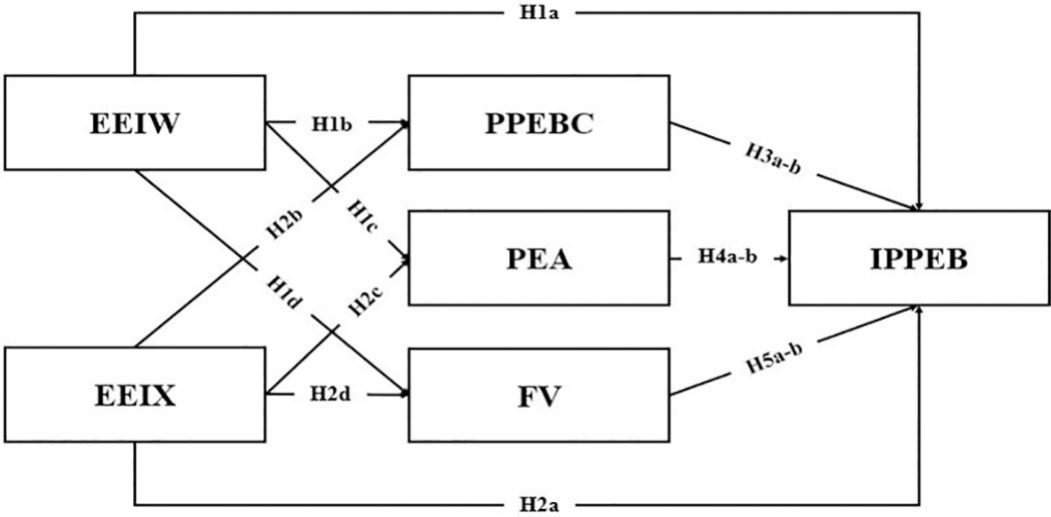
Model of predictors of the intention to participate in pro-environmental behavior.

According to a previous study, information about threats posed by the environment in the media increases individuals’ perceptions of the relevant topic and causes fear [66]. To protect their interests, individuals tend to take actions that reduce the fear of threats and eliminate the negative effects of threats [67]. A recent study found that exposure to information about environmental disasters provokes fear. Moreover, the increase in fear reinforces individuals’ pro-environmental intentions [17]. Social media users’ exposure to climate change-related videos increases their perceived fear [68], which induces their willingness to engage in pro-environmental behaviors [39]. Thus, Fig 1 indicates the hypothesis model.

H5a: FV mediates the relationship between EEIW and IPPEB.
H5b: FV mediates the relationship between EEIX and IPPEB.

## Research methods

### Research design

This study employed an empirical cross-sectional approach, conducting an open survey online from 1 January to 1 October 2022. The carefully designed questionnaire aimed to collect data from approximately 350 respondents aged 18–25, specifically targeting young adults whose social media usage is among the highest [69]. For the data collection, a convenience sample was utilized, which is recognized for its efficiency, speed, and cost-effectiveness in collecting nonprobability samples, and this method is also practical for generating hypotheses [70]. Drawing upon these advantages, previous studies have effectively employed convenience samples to investigate issues related to problematic social media use and consumer purchase behavior among social media users [71, 72].

### Data collection procedures

Data were collected between 1 January and 1 October 2022 through an online survey. Of the 365 potential respondents who received the survey link, 310 completed the questionnaire. Data cleaning is an essential step prior to data analysis. To eliminate abnormal sampling methods, this study combines three methods previously applied by scholars [73, 74]. The data cleaning process was as follows: (1) data samples that provided nonsense responses and unrelated and offensive open-ended answers were removed (N = 10), (2) “straight-lined” data samples were extracted; in other words, participants who intentionally selected the same answer within scale(s) were removed (N = 5), and (3) participants who completed the survey in an amount of time lower than one-third of the average level, the so-called “speeders,” were removed (N = 4). The final sample size included 291 participants.

Several studies have been conducted to evaluate the required sample size using G-Power 3.1 for quantitative-deductive design studies [75, 76]. Following the criteria of Faul et al. [77], the current study was configured with a power of 0.95, an effect size of 0.15, and five predictors. The results indicated that the minimum required sample size was 138. To estimate sample size for mediation models, the current study used Monte Carlo power analysis [78], a method widely used in the fields of media and psychology [79, 80]. Following the suggested minimum sample size, 150 individuals were required, and the analysis was conducted with 1000 and 2000 replications, with a confidence interval set at 95%. The results indicated that with a power of 0.95, the minimum sample size required was 228. In other words, the sample size of the current study (N = 291) is higher than the recommended sample size. Therefore, the sample is considered adequate.

### Data analysis methods

In this study, all data analysis was conducted using SPSS 22. The software was first used to assess construct reliability, convergent validity, discriminant validity, and Cronbach’s alpha [81]. It was also used for descriptive statistics and correlation analysis [82]. Hierarchical regression analysis was used to test the proposed direct effects hypotheses [83]. In addition, mediation hypotheses were tested using Hayes’ PROCESS macro for SPSS (20), followed by bootstrapping to obtain bias-corrected 95% confidence intervals for making statistical inferences about specific mediation effects [84].

### Measurement of variables

Six main measures (EEIW, EEIX, PPEBC, PEA, FV, and IPPEB) were carefully adjusted according to the following rules: First, measurements were translated from English to Chinese by two linguistic experts. Subsequently, all researchers reviewed the translated measures until they reached the same agreement on translation quality and accuracy [85]. Second, content and face validity methods were applied to readjust and remove the items [86]. Three specialists from the Department of Mass Communication were invited for an in-depth evaluation [85]. Finally, a pre-test was conducted with 10 volunteers recruited from the Beijing Institute of Graphic Communication. The participants were asked to provide suggestions for further adjustments to items that were unreadable or unclear [87]. The finalized questionnaire was adjusted by volunteers’ feedback and uploaded to a Chinese online survey platform “Wenjuanxing.” Wenjuanxing is known as a popular survey dissemination platform in China. This platform provides a sampling pool of nearly 260 million registered users in China [88]. Moreover, this approach has been used by numerous institutes and scholars to conduct studies in the Chinese context [89, 90]. The authors followed the ethical guidelines of the Beijing Institute of Graphic Communication. Permission to conduct this study was reviewed and approved by the Beijing Institute of Graphic Communication Ethics Committee (protocol code: L20210301). The written informed consent was provided at the beginning of the online questionnaire and the respondents were guaranteed confidentiality.

The measurements were evaluated using a five-point Likert scale; that is, participants were asked to answer from strongly disagree to strongly agree or very rarely to very frequently. EEIW and EEIX are measured as an index of how frequently an individual is exposed to the following five environment-related topics on WeChat and Xiaohongshu: (1) carbon dioxide on global warming; (2) rising sea level; (3) greenhouse effect; (4) news, reports, and climate experts; and (5) melting Arctic ice sheet. A composite measure was constructed for EEIW (M = 2.62, SD = 1.21, Cronbach’s α = 0.96) and EEIX (M = 2.34, SD = 1.23, Cronbach’s α = 0.98).

PPEBC was operationalized with four items derived from a previous study [91]. Examples of these statements are: (1) “With my financial ability, I am more inclined to buy environmentally friendly items” and (2) “If I want to, I can develop environmental habits.”. A composite measure was constructed (M = 3.90, SD = 0.86, Cronbach’s α = 0.90).

The four-item PEA measurement was adapted from an existing scale [21]. The respondents evaluated their beliefs about engaging in pro-environmental behaviors and indicated their level of agreement with the following questions using a 5-point Likert scale: Participation in pro-environmental behaviors is (1) enjoyable, (2) beneficial, (3) important, and (4) pleasant. A composite measure was constructed (M = 3.53, SD = 0.93, Cronbach’s α = 0.84).

Four items were derived from a prior study to observe FV [17]. This scale was used to evaluate the respondents’ awareness of the negative effects on the environment after being exposed to information on global warming in short-form video applications. Respondents answered about their level of agreement with questions using a 5-point Likert scale. Examples of these statements are: (1) “I am very afraid that I or my relatives and friends will become victims of global warming” and (2) “The impact of global warming has severely affected my future plan.”. A composite measure was constructed (M = 3.48, SD = 0.89, Cronbach’s α = 0.87).

The IPPEB scale was derived from previous research [92]. This scale assesses individuals’ willingness to participate in all possible actions aimed at safeguarding the environment. Based on the original scale, it was designed with three public and three private spheres of pro-environmental behaviors. Respondents indicated their level of agreement with questions using a 5-point Likert scale. Examples of these statements are (1) “Purchase green products and service whenever possible” and (2) “Remind and persuade friends or colleagues to protect the environment.”. A composite measure was constructed (M = 3.52, SD = 0.90, Cronbach’s α = 0.90).

To validate the measurement scales, this study conducted both convergent and discriminant validity tests to confirm the level of credibility and accuracy of each scale. First, the convergent validity tests followed the procedure suggested by Puriwat and Tripopsakul [93] and Mingwei et al. [94]. The current study examined a total of 28 items, including EEIW, EEIX, PPEBC, PEA, FV, IPPEB, and their loadings(see Table 1). The results indicated that the level of convergent validity was adequate, as the Average Variance Extracted (AVE) for all constructs ranged from 0.58 to 0.78.

**Table 1:** Reliability and validity of constructs.

Variables	Items	Estimate	CR	AVE	Cronbach’s Alpha
**EEIW**	EEIW1	0.86	0.95	0.711	0.96
	EEIW2	0.85			
	EEIW3	0.83			
	EEIW4	0.79			
	EEIW5	0.87			
**EEIX**	EEIX1	0.88	0.96	0.78	0.98
	EEIX2	0.89			
	EEIX3	0.90			
	EEIX4	0.89			
	EEIX5	0.87			
**PPEBC**	PPEBC1	0.73	0.98	0.58	0.90
	PPEBC2	0.73			
	PPEBC3	0.75			
	PPEBC4	0.83			
**PEA**	PEA1	0.83	0.98	0.65	0.84
	PEA2	0.86			
	PEA3	0.79			
	PEA4	0.81			
**FV**	FV1	0.72	0.95	0.58	0.87
	FV2	0.77			
	FV3	0.80			
	FV4	0.78			
**IPPEB**	IPPEB1	0.83	0.98	0.66	0.90
	IPPEB2	0.82			
	IPPEB3	0.81			
	IPPEB4	0.75			
	IPPEB5	0.82			
	IPPEB6	0.85			

* p<0.05, ** p<0.01, *** p<0.001; EEIW = exposure to environmental information on WeChat; EEIX = exposure to environmental information on Xiaohongshu; PPEBC = perceived pro-environmental behavior control; PEA = pro-environmental attitude; FV = fear of victimization; IPPEB = intention to participate in pro-environmental behavior

Following the methods of previous empirical studies, the Fornell-Larcker criterion has been widely accepted for testing discriminant validity [75, 93]. Therefore, this testing method was used in this study and the results are shown in Table 2. The Fornell-Larcker criterion suggests that the square root of the AVE values should be greater than the values of the correlations with other constructs [93, 94]. Thus, each construct demonstrated adequate discriminant validity.

**Table 2:** Fornell-Larcker criterion.

Variables	1	2	3	4	5	6
**EEIW**	**0.84**					
**EEIX**	0.76	**0.88**				
**PPEBC**	0.17	0.11	**0.76**			
**PEA**	0.36	0.25	0.49	**0.80**		.
**FV**	0.35	0.31	0.50	0.36	**0.76**	
**IPPEB**	0.36	0.31	0.65	0.62	0.48	**0.81**

* p<0.05, ** p<0.01, *** p<0.001; EEIW = exposure to environmental information on WeChat; EEIX = exposure to environmental information on Xiaohongshu; PPEBC = perceived pro-environmental behavior control; PEA = pro-environmental attitude; FV = fear of victimization; IPPEB = intention to participate in pro-environmental behavior

In the current study, data were collected through a cross-sectional survey. Therefore, it is essential to assess the presence of common method bias (CMB) [94]. Following the method of Podsakoff et al. [95], Harman’s one-factor test was applied. The results showed that the single factor accounted for 39.80%, which is below the recommended limit of 50%. Therefore, CMB does not appear to be a significant problem in this study.

## Results

### Descriptive data

A total of 291 valid responses were obtained. Table 3 shows the demographic characteristics of the participants. The participants were mostly women (N = 190, 65.3%). Moreover, most of the respondents were undergraduates (N = 191, 65.6%) or high school students (N = 57, 19.6%). Furthermore, the majority of participants’ ages ranged from 18 to 22 years (N = 248, 85.2%), followed by 23 to 25 years (N = 43, 14.8%). Approximately 61.5% of respondents were single (N = 179, 61.5%). The rest of the respondents were in relationships (N = 96, 33.0%) or married (N = 16. 5.5%). The results also reveal that most respondents’ monthly income ranged from 1,000 RMB to 6,999 RMB (N = 124, 42.6%), followed by 7,000 RMB to 14,000 RMB (N = 101, 34.7%), 14,000 RMB to 49,999 RMB (N = 55, 18.9%) and > 50,000 RMB (N = 11, 3.8%).

**Table 3:** Key demographic characteristics of the survey participants.

Variables	Item	Count	Percentage
**Gender**	Female	190	65.3%
	Male	101	34.7%
**Education level**	High school	57	19.6%
	Undergraduate	191	65.6%
	Postgraduates	43	14.8%
**Age**	18–22 years old	248	85.2%
	23–25 years old	43	14.8%
**Marital status**	Single	179	61.5%
	In a relationship	96	33.0%
	Married	16	5.5%
**Monthly income**	1,000–6,999 RMB	124	42.6%
	7000–14,000 RMB	101	34.7%
	14,000–49,999 RMB	55	18.9%
	50,000 < RMB	11	3.8%
	Total	291	100%

* p<0.05, ** p<0.01, *** p<0.001; EEIW = exposure to environmental information on WeChat; EEIX = exposure to environmental information on Xiaohongshu; PPEBC = perceived pro-environmental behavior control; PEA = pro-environmental attitude; FV = fear of victimization; and IPPEB = intention to participate in pro-environmental behavior

### Hypothesis testing

To test Hypotheses 1 and 2, this study used hierarchical regression analyses with IPPEB, PPEBC, PEA, and FV as dependent variables. The decision to employ this specific analysis in the current study is motivated by two reasons. Firstly, many scholars have utilized such an approach to examine the unique variance contributed by each additional predictor while simultaneously controlling for the effects of previously entered predictors (e.g., knowledge-sharing behavior, prosocial behavior, and engagement in physical activities) [96–98]. Secondly, a prior study stressed that employing hierarchical regression enhanced the precision of the analysis, resulting in more stable and reliable outcome values [99]. In the current study, gender, education, and income were entered in the first block as controlling confounders. EEIW and EEIX were entered in the second block. The effects of EEIW on IPPEB (β = 0.30, t = 6.90, p <0.001), PPEBC (β = 0.19, t = 4.40, p <0.001), PEA (β = 0.30, t = 6.88, p <0.001), and FV (β = 0.27, t = 6.46, p <0.001) were significant. Therefore, H1 (a–d) was supported. The effects of EEIX on IPPEB (β = 0.25, t = 6.10, p <0.001), PPEBC (β = 0.14, t = 3.31, p <0.01), PEA (β = 0.22, t = 4.93, p <0.001), and FV (β = 0.23, t = 5.39, p <0.001) were significant. Therefore, H2 (a–d) was supported. Table 4 presents the results of the direct effect.

**Table 4:** Summary of direct effects.

Direct effect	Effect	t value	p value	Result
EEIW→IPPEB	0.30	6.90	p <0.001	Supported
EEIW→PPEBC	0.19	4.40	p <0.001	Supported
EEIW→PEA	0.30	6.88	p <0.001	Supported
EEIW→FV	0.27	6.46	p <0.001	Supported
EEIX→IPPEB	0.25	6.10	p <0.001	Supported
EEIX→PPEBC	0.14	3.31	p <0.01	Supported
EEIX→PEA	0.22	4.93	p <0.001	Supported
EEIX→FV	0.23	5.39	p <0.001	Supported

* p<0.05, ** p<0.01, *** p<0.001; EEIW = exposure to environmental information on WeChat; EEIX = exposure to environmental information on Xiaohongshu; PPEBC = perceived pro-environmental behavior control; PEA = pro-environmental attitude; FV = fear of victimization; IPPEB = intention to participate in pro-environmental behavior

**Fig 2:**
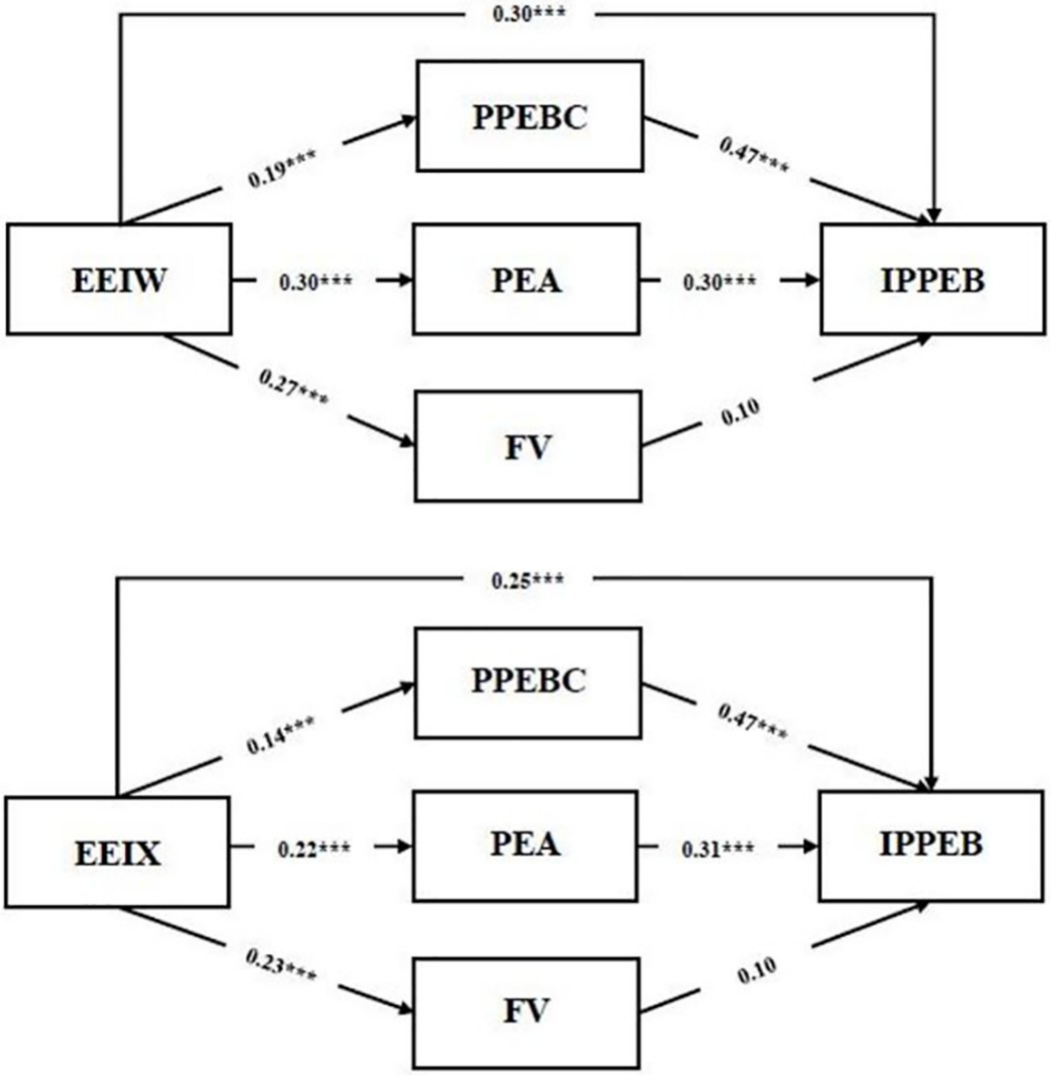
Model of EEIW and EEIX for IPPEB, mediated by PPEBC, PEA, and FV.

The Hayes’ PROCESS macro (model 4) was used to test the mediating hypotheses. This study used bootstrapping to obtain bias-corrected 95% confidence intervals to make statistical inferences about specific indirect effects. Fig 2 indicates the standardized coefficients and significance for each path in the hypothesized models of EEIW and EEIX for IPPEB.

The following results show the mediating effects of EEIW on IPPEB. In the first mediation model, PPEBC had a positive effect on IPPEB (β = 0.47, t = 9.03, p < 0.001), and the indirect effect was significant (β = 0.10, t = 6.62, p < 0.001, 95% CI [0.03, 0.12]). The results of the second mediation model indicated that PEA had a positive effect on IPPEB (β = 0.30, t = 6.53, p < 0.001), and the indirect effect was significant (β = 0.10, t = 6.62, p < 0.001, 95% CI [0.05, 0.13]). The results of the third mediation model showed that FV did not have a significant effect on IPPEB (β = 0.10, t = 2.13, p > 0.05), and the indirect effect was insignificant (β = 0.03, t = 6.63, p > 0.05, 95% CI [-0.01, 0.06]). Therefore, H3a and H4a were supported, whereas H5a was rejected. The mediating effects on the relationship between EEIX and IPPEB indicated that the first mediation model demonstrated that PPEBC had a positive effect on IPPEB (β = 0.47, t = 9.18, p < 0.001), and the indirect effect was significant (β = 0.05, t = 5.54, P < 0.001, 95% CI [0.01, 0.09]). The second mediation model demonstrated that PEA had a positive effect on IPPEB (β = 0.31, t = 6.90, p < 0.001), and the indirect effect was significant (β = 0.06, t = 5.54, p < 0.001, 95% CI [0.02, 0.10]). Finally, the results of the third mediation model indicated that FV did not have a significant effect on IPPEB (β = 0.10, t = 2.01, p > 0.05), and the indirect effect was insignificant (β = 0.02, t = 5.54, p > 0.05, 95% CI [-0.01, 0.05]). Thus, H3b, H4b and H5b were supported. These indirect effects are summarized in Table 5.

**Table 5:** Summary of indirect effects.

Indirect effect	Effect	t value	p value	Lower	Upper	Result
EEIW→PPEBC→IPPEB	0.10	6.62	p <0.001	0.03	0.12	Supported
EEIW→PEA→IPPEB	0.10	6.62	p <0.001	0.05	0.13	Supported
EEIW→FV→IPPEB	0.03	6.62	p <0.001	-0.01	0.06	Rejected
EEIX→PPEBC→IPPEB	0.05	5.54	p <0.001	0.01	0.09	Supported
EEIX→PEA→IPPEB	0.06	5.54	p <0.001	0.02	0.10	Supported
EEIX→FV→IPPEB	0.02	5.54	p <0.001	-0.01	0.05	Rejected

* p<0.05, ** p<0.01, *** p<0.001; EEIW = exposure to environmental information on WeChat; EEIX = exposure to environmental information on Xiaohongshu; PPEBC = perceived pro-environmental behavior control; PEA = pro-environmental attitude; FV = fear of victimization; IPPEB = intention to participate in pro-environmental behavior

## Discussion

Guided by the TPB, this study investigated the effects of environmental information exposure on pro-environmental behavior intentions of Chinese young adults (18–25 years old). The main objective of this study was to examine the direct effect. In addition, the indirect effects of cognitive (PPEBC), psychological (PEA), and emotional (FV) factors on this relationship among the young adults were thoroughly investigated.

In terms of direct effects, this study found that the EEIW and EEIX of Chinese young adult users positively affected PPEBC, PEA, and FV. In terms of direct effects on the relationship between EEIW, EEIX, and IPPEB, the findings are in line with those of previous studies that claimed that exposure to information in the media amplifies individuals’ adaptation to eco-friendly behaviors [54, 55]. Additionally, the EEIW and EEIX in Chinese young adult users are positively associated with PPEBC, which supports the idea that PPEBC is positively affected by social media information exposure [49, 60]. For instance, a previous study claimed that exposure to environmental content on social media largely enhanced individuals’ pro-environmental behavior control [11]. Furthermore, higher EEIW and EEIX levels led to higher PEA levels in Chinese young adults. These results are in line with those of previous studies demonstrating that frequent exposure to environmental information has a positive effect on individuals’ attitudes toward environmental protection [56]. Finally, a prior study explained that the more individuals are exposed to disaster-related information, the more their FV is induced. Moreover, this study found a direct relationship between EEIW, EEIX, and FV [17].

In terms of mediated effects, PPEBC mediated both EEIW and EEIX effects on IPPEB (H3a–b). This was consistent with a previous study that found that the relationship between students’ environmental information exposure and environmental behaviors was mediated by PPEBC [100]. In terms of the mediating effect of PEA on the relationship between EEIW, EEIX, and IPPEB (H4a–b), the results showed that PEA played a mediating role in such relationships. This finding is in line with that of a previous study indicating that exposure to climate change-related issues positively affects attitudes toward environmental protection [62]. Ultimately, PEA increases individuals’ participation in pro-environmental behaviors [63]. Finally, FV did not mediate the relationship between EEIW, EEIX, and IPPEB (H5a–b), which is contrary to the findings of previous studies. For example, previous findings have shown that high exposure to climate change-related topics through online videos can influence individuals’ perceived fear [68] and further enhance their willingness to mitigate climate change [39]. In addition, a previous study suggested that frequent exposure to fearful information increased individuals’ willingness to protect the environment [57]. Similarly, Yang and Shi [101] claimed that among the channels, new media such as WeChat and microblogs are considered as major platform to share air pollution to public, and have an huge impact on individuals’ cognition. Moreover, study has shown that fearful environmental content such as smog crisis-related information has been broadly exposed to Chinese social media users, which has further increase their engagement in the China smog crisis, both on SNSs and on the ground (pro-environmental behavior) [102]. However, in China, WeChat and Xiohongshu had high concentrations of positive environmental information. For instance, a key strategy of environmental campaigns on WeChat stresses that creating more positive content may lead users to think positively and feel satisfied [103]. Similarly, green advertising on Xiaohongshu’s aims and objectives has emphasized that the media should encourage more users to follow media influencers’ pro-environmental behaviors and participate in in-person events. Such events may inspire joy and connection among users (e.g., sharing second-hand clothes and making clothes using recycled materials), which further induces their engagement in eco-friendly behaviors [104]. Chinese WeChat and Xiaohongshu users being fed with less threatening environmental information is assumed to be the main reason for their IPPEB failure.

Noticeably, different social platforms in China (Xiaohongshu and WeChat) demonstrated the same media effect on the IPPEB in Chinese young adults. However, EEIX shows weaker direct effects on PEA (β = 0.22, p <0.001) than EEIW. A previous study argued that discussions on social media play a major role in attitude formation [105]. Another study indicated that Twitter users who actively engaged in online discussions on climate change showed a positive attitude toward it [106]. WeChat is one of the most used platforms that provide users with the opportunity to exchange and acquire information with a higher volume [107]. This may be a possible reason why EEIW has a stronger effect on PEA than EEIX.

## Theoretical and practical implications

In terms of theoretical contributions, the current study has expanded our knowledge in several ways. First, in response to criticisms of the TPB model and to enhance its explanatory capacity, this study is one of the earliest to extend the TPB model with fear of victimization. Therefore, it has enhanced the theory’s applicability and explanatory power when investigating the pro-environmental behaviors of Chinese young adults. Second, this study has enriched the theoretical framework by providing a deeper understanding. Specifically, it has provided empirical evidence that cognitive, psychological, and emotional factors act as mediating mechanisms in the relationship between environmental information exposure on both WeChat and Xiaohongshu and the intention to participate in pro-environmental behavior among Chinese young adults. Third, the findings of this study support the idea that both cognitive and psychological factors mediate such a relationship. These results align with previous studies [49, 58–63]. In China, short-form video applications, one of the most popular new media platforms, have become essential mediums for disseminating a wide range of information [108]. Therefore, we strongly encourage future studies to explore the role of short-form video platforms and their different types of content (e.g., official and user-generated content) in shaping pro-environmental behavior among young adults, as well as how the TPB can be applied in these contexts.

It is essential to provide policy and management implications in social science research. Based on the current findings, this study found that there are no significant differences in the effects of different social media platforms (Wechat and Xiaohongshu) in China on intention to participate in pro-environmental behavior. Therefore, policy makers should focus on providing more effective pro-environmental promotion on social media to encourage individuals to engage in pro-environmental behavior. A previous study showed that WeChat is known for providing the latest information, which helps individuals keep up with current social events However, false information and excessive notifications affect the quality of information [46]. In addition, a previous study showed that the efficiency of information has a direct effect on climate-related information avoidance [109]. Therefore, social media managers should carefully consider the quality of environmental information and set appropriate notification settings to satisfy users’ information needs and willingness to accept the information. Moreover, this study also suggests WeChat and Xiaohongshu incorporate more features of interpersonal communication. By doing so, the platforms can foster a greater sense of community engagement and support, leading to an increase in pro-environmental actions.

## Limitations

The following limitations should be noted for future studies. First, this study was conducted using a cross-sectional design. Therefore, the current study is limited in establishing the causal effects of environmental information exposure on WeChat and Xiaohongshu on Chinese young adults’ intention to engage in pro-environmental behaviors. In order to conduct comprehensive investigations, it is highly recommended that future studies adopt longitudinal or experimental designs.

Second, the measure of fear of victimization was operationalized using four items derived from Shah et al. [17]. We acknowledge that the limited number of items may not accurately measure the fear experienced by Chinese young adults when exposed to information about global warming and its negative effects on the environment. Therefore, more precise scales are recommended and their reliability and validity should be tested in the Chinese context. Finally, this study did not consider cognitive bias factors such as optimistic bias and status quo bias. Therefore, future research should consider the aforementioned factors to investigate their mediating mechanisms in the context of environmental information exposure on both WeChat and Xiaohongshu and intention to participate in pro-environmental behavior among Chinese young adults.

## Conclusions

The current study examined the relationship between exposure to environmental information on WeChat and Xiaohongshu and the intention to engage in pro-environmental behavior among Chinese young adults aged 18–25. To our knowledge, this is the first study to uncover the direct and mediated effects of cognitive, psychological, and emotional factors on the intention to engage in pro-environmental behavior among Chinese young adults. This finding has significant implications for Chinese ecology and environmental departments and related professionals, as it allows for the development of more effective social media promotion campaigns. Encouraging greater participation in pro-environmental activities among young adults can help mitigate climate damage and contribute to climate restoration efforts.

## Supporting information

S1 Table Measurement of variables. (DOCX)

S2 Table Key demographic characteristics of the survey participants. (DOCX)

S3 Table Correlations between key variables. (DOCX)

S4 Table Summary of direct effects. (DOCX)
