# Supplementary material for: The effect of social media environmental information exposure on the intention to participate in pro-environmental behavior
Source: PLoS One. 2023 Nov 16;18(11):e0294577. doi: 10.1371/journal.pone.0294577 (PMC10653508; doi:10.1371/journal.pone.0294577)
Supplement: S1 Table — (DOCX) [file pone.0294577.s001.docx]

**Table1. Measurement of variables**

| **Item** | **M** | **SD** | **α** |
| --- | --- | --- | --- |
| EEIW1 | 2.64 | 1.27 | 0.96 |
| EEIW2 | 2.53 | 1.25 |  |
| EEIW3 | 2.60 | 1.25 |  |
| EEIW4 | 2.78 | 1.24 |  |
| EEIW5 | 2.55 | 1.26 |  |
| EEIX1 | 2.31 | 1.25 | 0.98 |
| EEIX2 | 2.32 | 1.30 |  |
| EEIX3 | 2.36 | 1.28 |  |
| EEIX4 | 2.33 | 1.31 |  |
| EEIX5 | 2.35 | 1.31 |  |
| PPEBC1 | 3.70 | 1.00 | 0.90 |
| PPEBC2 | 3.71 | 1.10 |  |
| PPEBC3 | 3.39 | 1.04 |  |
| PPEBC4 | 3.13 | 1.12 |  |
| PEA1 | 3.70 | 1.00 | 0.84 |
| PEA2 | 3.71 | 1.09 |  |
| PEA3 | 3.40 | 1.04 |  |
| PEA4 | 3.13 | 1.18 |  |
| FV1 | 3.70 | 1.00 | 0.87 |
| FV2 | 3.71 | 1.09 |  |
| FV3 | 3.40 | 1.04 |  |
| FV4 | 3.13 | 1.12 |  |
| IPPEB1 | 3.61 | 1.10 | 0.90 |
| IPPEB2 | 3.53 | 1.10 |  |
| IPPEB3 | 3.34 | 1.13 |  |
| IPPEB4 | 3.56 | 1.11 |  |
| IPPEB5 | 3.54 | 1.11 |  |
| IPPEB6 | 3.55 | 1.08 |  |
