# Supplementary material for: The effect of social media environmental information exposure on the intention to participate in pro-environmental behavior
Source: PLoS One. 2023 Nov 16;18(11):e0294577. doi: 10.1371/journal.pone.0294577 (PMC10653508; doi:10.1371/journal.pone.0294577)
Supplement: S2 Table — (DOCX) [file pone.0294577.s002.docx]

**Table 2. Key demographic characteristics of the survey participants.**

| **Variables** | **Item** | **Count** | **Percentage** |
| --- | --- | --- | --- |
| Gender | Female | 190 | 65.3% |
|  | Male | 101 | 34.7% |
| Education level | High school | 57 | 19.6% |
|  | Undergraduate | 191 | 65.6% |
|  | Postgraduates | 43 | 14.8% |
| Age | 18–22 years old | 248 | 85.2% |
|  | 23–25 years old | 43 | 14.8% |
| Marital status | Single | 179 | 61.5% |
|  | In a relationship | 96 | 33.0% |
|  | Married | 16 | 5.5% |
| Monthly income | 1,000–6,999 RMB | 124 | 42.6% |
|  | 7000–14,000 RMB | 101 | 34.7% |
|  | 14,000–49,999 RMB | 55 | 18.9% |
|  | 50,000 < RMB | 11 | 3.8% |
|  | Total | 291 | 100% |
