# Supplementary material for: The effect of social media environmental information exposure on the intention to participate in pro-environmental behavior
Source: PLoS One. 2023 Nov 16;18(11):e0294577. doi: 10.1371/journal.pone.0294577 (PMC10653508; doi:10.1371/journal.pone.0294577)
Supplement: S3 Table — (DOCX) [file pone.0294577.s003.docx]

**Table 3. Correlations between key variables.**

| **Variables** | **1** | **2** | **3** | **4** | **5** |
| --- | --- | --- | --- | --- | --- |
| EEIW | – |  |  |  |  |
| EEIX | 0.76** | – |  |  |  |
| PPEBC | 0.22** | 0.14* | – |  |  |
| PEA | 0.36** | 0.25** | 0.53** | – |  |
| FV | 0.35** | 0.31** | 0.52** | 0.36** | – |
| IPPEB | 0.36** | 0.31** | 0.69** | 0.62** | 0.48** |
| Mean | 2.62 | 2.33 | 3.90 | 3.53 | 3.50 |
| SD | 1.16 | 1.23 | 0.86 | 0.93 | 0.90 |
