# Supplementary material for: The effect of social media environmental information exposure on the intention to participate in pro-environmental behavior
Source: PLoS One. 2023 Nov 16;18(11):e0294577. doi: 10.1371/journal.pone.0294577 (PMC10653508; doi:10.1371/journal.pone.0294577)
Supplement: S4 Table — (DOCX) [file pone.0294577.s004.docx]

**Table 4. Summary of direct effects**

| **Direct effect** | **Effect** | **p value** | **Result** |
| --- | --- | --- | --- |
| EEIW→IPPEB | 0.30 | p <0.001 | Supported |
| EEIW→PPEBC | 0.19 | p <0.001 | Supported |
| EEIW→PEA | 0.30 | p <0.001 | Supported |
| EEIW→FV | 0.27 | p <0.001 | Supported |
| EEIX→IPPEB | 0.25 | p <0.001 | Supported |
| EEIX→PPEBC | 0.14 | p <0.01 | Supported |
| EEIX→PEA | 0.22 | p <0.001 | Supported |
| EEIX→FV | 0.23 | p <0.001 | Supported |
